# Detaching Photosensitive Nanoparticles from Cells Prevents Them from Remaining in Cells after Photoporation

**DOI:** 10.1002/advs.77816

**Published:** 2026-09-25

**Authors:** Tao Lu, Mina Nikolić, Baihao Huang, Deep Punj, Herlinde De Keersmaecker, Thibaut Van Acker, Wenjia Xie, Florian Vanlauwe, Phillip Blondeel, Liesl De Graeve, Félix Sauvage, Ranhua Xiong, Chaobo Huang, Frank Vanhaecke, Kevin Braeckmans, Stefaan C. De Smedt

**Affiliations:** ^1^ State Key Laboratory for Development and Utilization of Forest Food Resources Joint Laboratory of Advanced Biomedical Materials (NFU‐UGent) Jiangsu Co‐Innovation Center of Efficient Processing and Utilization of Forest Resources Nanjing Forestry University Nanjing China; ^2^ Laboratory of General Biochemistry and Physical Pharmacy Faculty of Pharmaceutical Sciences Ghent University Ghent Belgium; ^3^ Atomic and Mass Spectrometry – A&MS research group Department of Chemistry Faculty of Sciences Ghent University Ghent Belgium; ^4^ Ghent Light Microscopy Core Ghent University Ghent Belgium; ^5^ Tissue Regeneration and Organ Printing (TROP) Research Center Department of Human Structure and Repair Ghent University Ghent Belgium; ^6^ Department of Plastic and Reconstructive Surgery Ghent University Hospital Ghent Belgium

**Keywords:** gold nanoparticles, intracellular delivery, phenylboronic acid, photoporation

## Abstract

Efficient and safe intracellular delivery of macromolecules, like nucleic acids, remains a major challenge in ex vivo cell engineering. While photoporation is a highly attractive method for cytosolic delivery of (macro)molecules, photosensitizers remaining in the photoporated cells may pose safety concerns, especially when the cells are engineered for therapeutic use. In this study we introduce the concept of “detachable photosensitizers” using gold nanoparticles (AuNPs) as photosensitizer. We demonstrate that phenylboronic acid (PBA)‐functionalized AuNPs can be anchored to membranes of HeLa cells and Jurkat cells, thereby enabling the efficient intracellular delivery of dextrans and mRNA upon pulsed laser irradiation (photoporation). Subsequently, the PBA‐AuNPs can be detached from the cells “on‐demand” by adding ATP to the cell medium, as *cis‐*diol groups in ATP displace the boronate ester bonds between the PBA‐AuNPs and cell‐surface. ICP‐MS analysis demonstrated efficient detachment, with the average cell‐associated gold mass per viable cell falling below the theoretical mass of a single intact 80 nm AuNP. This “attach‐and‐detach” strategy offers a robust and effective approach to addressing safety concerns associated with the use of plasmonic nanomaterials as sensitizers for photoporation, and holds significant potential for the safe manufacturing of engineered cells in the context of cell therapy.

## Graphical abstract

Phenylboronic acid‐functionalized gold nanoparticles reversibly attach to cell‐surface glycans, enhancing photoporation‐mediated intracellular delivery. After irradiation, ATP competitively disrupts the boronic ester interactions and removes cell‐associated nanoparticles, markedly reducing residual gold. This “attach‐and‐detach” strategy combines efficient cell engineering with improved post‐treatment material removal, offering a safer route toward standardized ex vivo cell manufacturing.

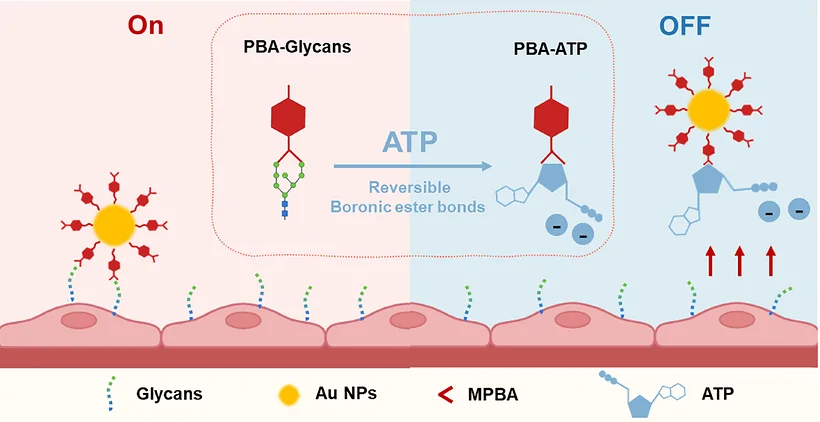

## 1 Introduction

Efficient intracellular delivery of plasma membrane‐impermeable biomolecules, including nucleic acids and proteins, remains a major challenge in both cell biology and applied research fields such as cell engineering [1] and drug delivery [2]. Current intracellular delivery methods can be broadly categorized into two main approaches: nanocarrier‐mediated delivery and cell membrane disruption‐based delivery [3]. In nanocarrier‐mediated approaches, exogenous cargos are typically encapsulated within viral or nonviral vectors (e.g., lipid nanoparticles as used in the mRNA COVID‐19 vaccines) that enter cells via endocytosis or via membrane fusion [4]. Following endocytic uptake, a substantial fraction of these nanocarriers becomes trapped within endosomal and lysosomal compartments, severely limiting cytosolic cargo availability. Although a wide variety of carriers have been developed, there remains room for improving their delivery efficiency, e.g., through enhancing the extent of endosomal escape, and cellular safety [5, 6]. Moreover, most nanocarriers are optimized for a specific class of cargo, which limits their general applicability.

Cell membrane disruption‐based methods create transient pores in the plasma membrane through either chemical, mechanical, or physical stimuli, thereby enabling “direct” cytosolic entry of exogenous cargos, thus bypassing their sequestration in endosomal and lysosomal compartments. Among them, electroporation is the oldest [7] and most widely used cell membrane disruption‐based method for intracellular delivery [8, 9]. It is suitable to deliver diverse types of molecules into various cell types through brief electric pulses that temporarily enhance membrane permeability [10, 11]. Despite its widespread use, electroporation remains, however, associated with significant limitations, including perturbation of cellular homeostasis and pronounced loss of cell viability [12, 13].

As a gentler alternative, “nanoparticle‐sensitized photoporation” has emerged as a promising technique. This method involves incubating cells with light‐absorbing nanoparticles, such as gold nanoparticles (AuNPs), iron oxide nanoparticles, or polydopamine nanoparticles, followed by irradiation of the cells with nano‐ or picosecond laser pulses [14, 15, 16, 17]. Upon laser exposure, the nanoparticles rapidly convert light energy into heat. At sufficient laser fluences, localized heating leads to the formation of vapor nanobubbles (VNBs) which expand and collapse near the cell membrane, thereby generating mechanical forces that transiently permeabilize the membrane and allow cargo entry [18, 19, 20, 21]. Over the past decade, this technique has been successfully applied by our group to deliver a wide range of molecules into both adherent and suspension cells [22, 23, 24].

However, a key limitation may restrict the broader application of nanoparticle‐sensitized photoporation in cell engineering for clinical purposes. Indeed, to photoporate cell membranes, cells must be incubated with the photosensitive nanoparticles for a certain time to allow sufficient nanoparticle attachment to the plasma membrane, prior to laser irradiation [25]. While this attachment is necessary to achieve effective photoporation, it simultaneously provides ample opportunity for the nanoparticles to internalize in the cells via endocytosis. This uptake should, however, be avoided when the ex vivo engineered cells have to be injected into patients; indeed, (residues of) photosensive nanoparticles in the photoporated cells may perturb cell functionality or raise long‐term safety concerns [26, 27, 28, 29, 30].

Avoiding the entrance of “foreign” materials in cells is a long lasting challenge in most diverse technologies in which cell culturing and/or cell engineering are involved. E.g., the production of “cell sheets” [31, 32], for the purpose of regenerative medicine, needs appropriate methods to gently detach the cells from their supporting material surfaces, without compromising cell surface integrity, viability and functionality, and avoiding the uptake of cell supporting material by the cells. As we recently reviewed, diverse strategies have studied to obtain ‘switchable’ interfaces that enable non‐invasive cell detachment, including the use of external physical stimuli (like light, temperature), chemical and biological stimuli [33, 34, 35, 36]. Inspired by this earlier research on how to “safely release” cells from surfaces on which they are cultured, we questioned whether strategies could be defined which allow to remove/detach (photosensitive) nanoparticles from the surface of cells, immediately following photoporation of the cells, this to avoid uptake of the nanoparticles by the cells.

**FIGURE 1:**
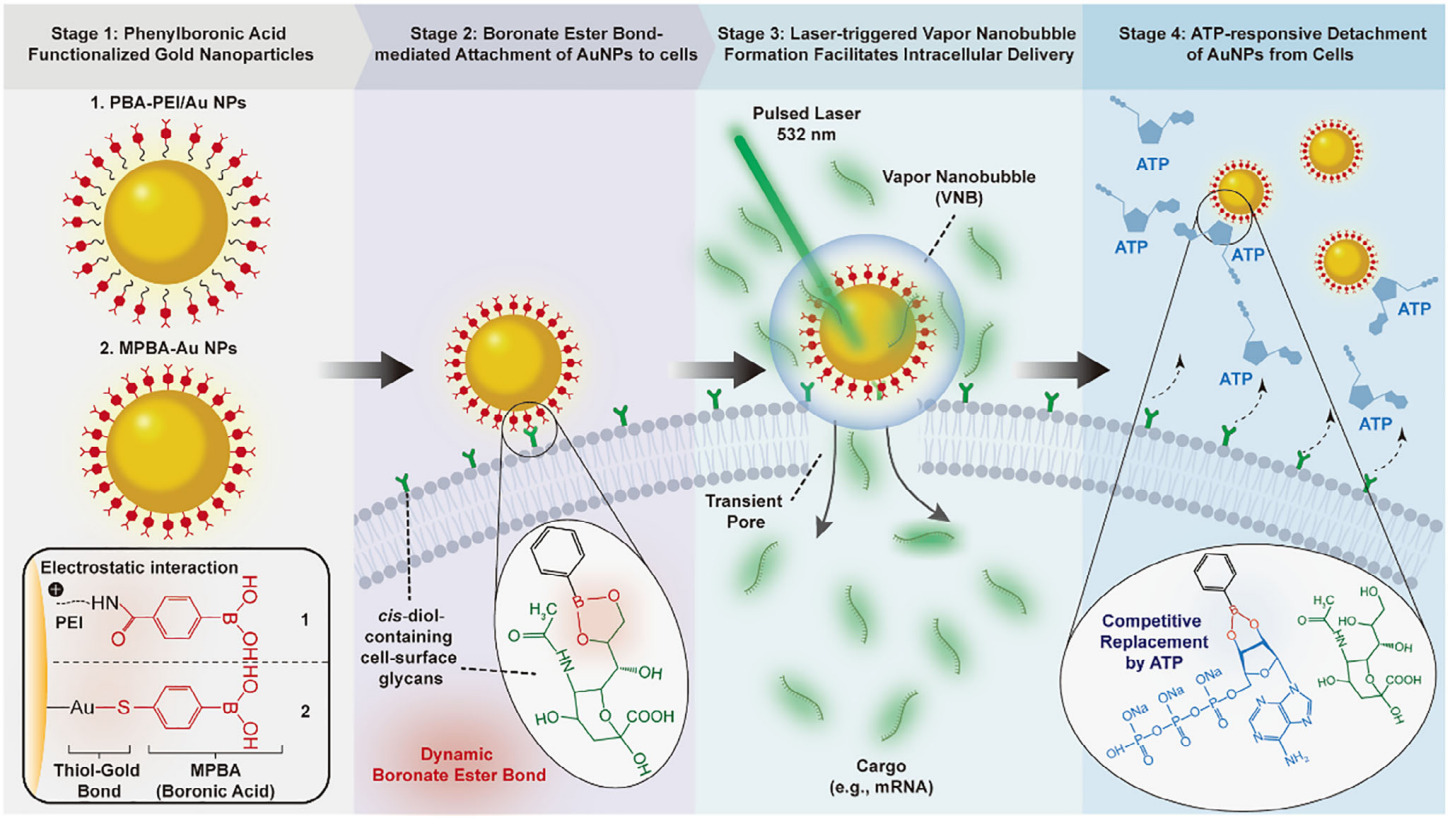
Schematic illustration of the phenylboronic acid (PBA) based concept to attach/detach gold nanoparticles (AuNPs) to/from cells for the purpose of photoporation. **Stage 1**: two strategies for PBA‐surface modification of AuNPs were considered. (i) Electrostatic assembly: the adsorption of cationic PBA‐PEI polymer onto the surface of negatively charged citrate‐stabilized AuNPs. (ii) Covalent conjugation: the functionalization of the gold surface with 4‐mercaptophenylboronic acid (MPBA), leveraging the formation of robust gold‐thiol (Au‐S) covalent bonds. **Stage 2**: PBA‐mediated association with *cis*‐diol‐containing cell‐surface glycans and glycoconjugates. **Stage 3**: Photoporation‐triggered vapor nanobubble‐mediated intracellular delivery of cargo. Generation of transient vapor nanobubbles (VNBs) upon pulsed laser irradiation, creating transient pores for the delivery of cargo (e.g., mRNA). **Stage 4**: ATP‐induced detachment of the AuNPs, ensuring minimal AuNP retention in the cells.

In this work, as illustrated in Figure 1, we introduce a strategy to “attach/detach” photosensitive gold nanoparticles (AuNPs) to/from the surface of cells, this to improve the performance and safety of cell engineering by photoporation. Two surface modification strategies are explored to functionalize the surface of AuNPs with phenylboronic acid (PBA): (i) the electrostatic assembly of cationic PBA‐PEI on the negatively charged surface of the citrate‐AuNPs and (ii) the covalent conjugation of 4‐mercaptophenylboronic acid (MPBA) via robust gold‐thiol (Au‐S) bonds, respectively. We evaluate whether thus functionalized AuNPs can be associated with *cis*‐diol‐containing cell‐surface glycans and glycoconjugates through the formation of boronate ester bonds [37, 38, 39, 40, 41]. Next, we study whether, upon nanosecond pulsed laser irradiation, the cell membrane‐attached AuNPs can trigger the formation of VNBs, thereby creating transient pores (photoporation) to facilitate the intracellular delivery of cargo. Subsequently, we study whether the AuNPs can be detached from the cells through competitive binding of ATP to the PBA moieties and to what extent this prevents AuNPs from remaining in the cells after photoporation.

## 2 Results and Discussion

### 2.1 Synthesis and Characterization of Phenylboronic Acid Functionalized Gold Nanoparticles

**FIGURE 2:**
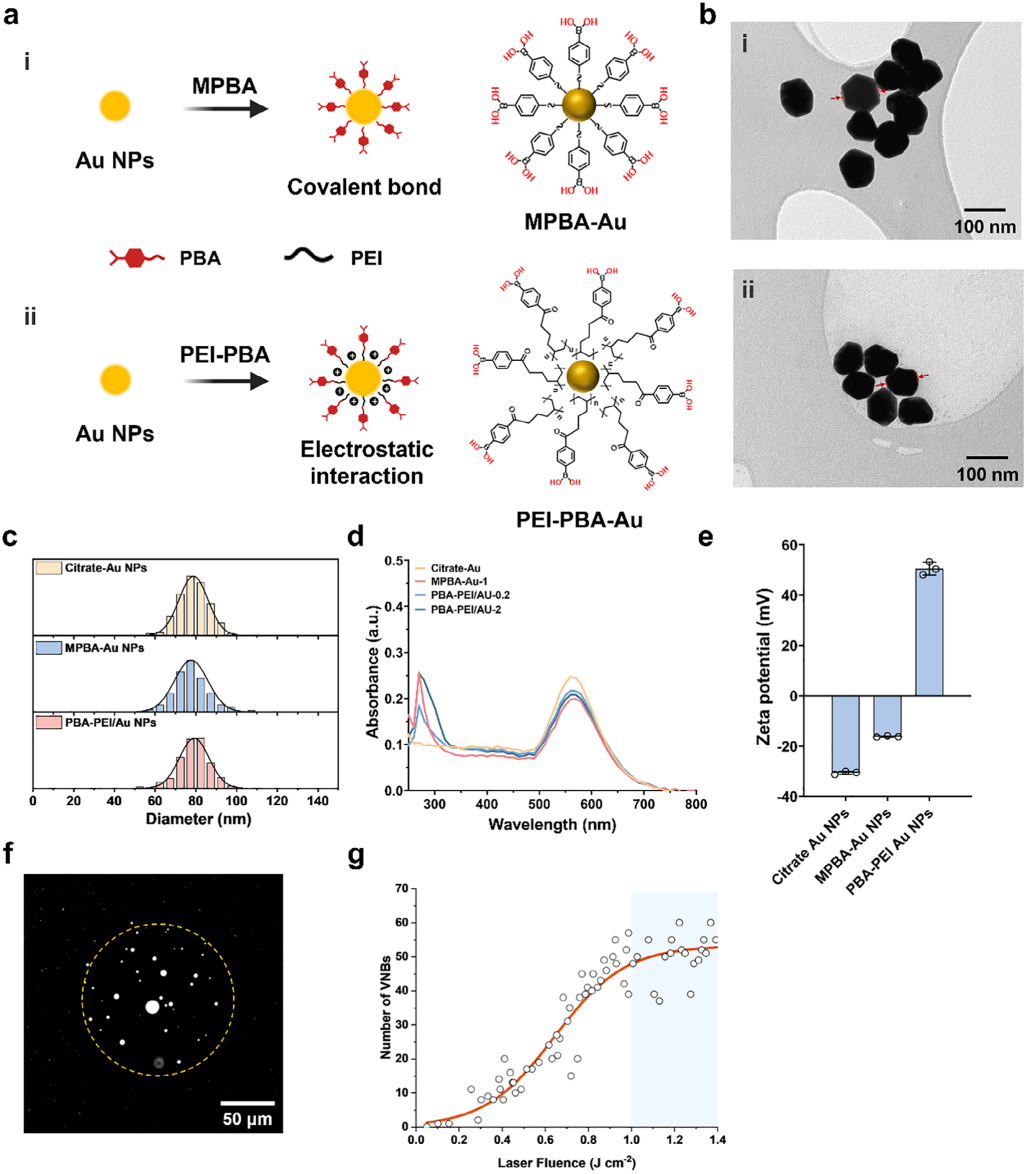
(a) Illustration of the strategies used for surface modification of the AuNPs: electrostatic adsorption using PBA‐PEI and covalent anchoring using MPBA. (b) Corresponding TEM images of PBA‐PEI/AuNPs and MPBA‐AuNPs showing preserved morphology after surface modification. The scale bar is 100 nm. (c) Hydrodynamic size distribution of the modified AuNPs. (d) UV–vis absorption spectra showing the plasmon resonance peak (∼560 nm) and the phenylboronic acid‐related peak (∼270 nm). (e) Zeta potential measurements illustrating distinct surface charges. (f) Representative dark‐field images visualizing VNB formation upon nanosecond laser irradiation of MPBA‐AuNPs. The scale bar is 50 µm. (g) Determination of the laser fluence threshold for VNB generation by MPBA‐AuNPs, defined as the fluence at which 90% of the AuNPs in the laser spot produce VNBs.

Two distinct strategies were developed to functionalize AuNPs with PBA moieties (Figure 2a). The first strategy was based on ionic interactions, where (positively charged) polyethylenimine‐phenylboronic acid (PBA‐PEI) was assembled onto negatively charged citrate‐capped AuNPs (Figure 2a(i)). The second approach relied on covalent surface anchoring, in which 4‐mercaptophenylboronic acid (MPBA) molecules were conjugated to AuNPs via Au─S bonds (Figure 2a(ii)). To avoid undesired aggregation of the AuNPs, the concentration of both surface modifiers was carefully optimized. Indeed, excessive amounts of either PBA‐PEI or MPBA induced nanoparticle aggregation, which might be attributed to charge neutralization of the AuNPs and the consequent reduction of electrostatic repulsion (data not shown). The optimal concentrations were determined to be approximately 0.300 ng µL^−1^ (13.3 nm) for PBA‐PEI and 0.154 ng µL^−1^ (1 µm) for MPBA. The large difference in molar concentrations can be attributed to the high charge density of PEI, which enables efficient displacement of citrate ligands and rapid reduction of electrostatic stabilization, thereby destabilizing citrate‐capped AuNPs at much lower concentrations.

Transmission electron microscopy (TEM) images confirmed that both PBA‐PEI/AuNPs and MPBA‐AuNPs retained a spherical morphology with a diameter of approximately 80 nm after surface functionalization, showing no visible signs of aggregation or morphological alteration compared to unmodified citrate‐AuNPs (Figure 2b). This was further supported by dynamic light scattering (DLS) measurements, which confirmed narrow and consistent size distributions across all formulations (Figure 2c). UV–vis absorption spectroscopy provided additional insights into surface modification and colloidal stability (Figure 2d). All AuNPs exhibited a characteristic localized surface plasmon resonance (LSPR) peak around 560 nm, with no significant red or blue shift after PBA modification, further providing evidence to the absence of aggregation. A slight decrease in absorbance intensity was observed compared to unmodified AuNPs, likely due to inevitable particle loss during repeated centrifugal washing steps. Notably, an additional absorbance peak emerged around 270 nm for both PBA‐PEI/AuNPs and MPBA‐AuNPs, which corresponds to the phenylboronic acid moiety. This peak was absent in citrate‐AuNPs and increased proportionally with the degree of surface modification, further validating successful surface functionalization of the AuNPs.

Surface charge measurements by zeta potential analysis confirmed distinct changes in particle surface properties upon modification. Citrate‐AuNPs exhibited a typical negative charge of around −30 mV. MPBA‐functionalized AuNPs displayed a partial shift toward neutrality (approximately −18 mV), which suggests partial displacement or shielding of the citrate layer without full charge reversal. In contrast, PBA‐PEI/AuNPs exhibited a strong charge inversion, reaching +50 mV due to the highly cationic nature of the PEI backbone. These results reflect the different surface binding mechanisms and charge modulation capabilities of the two PBA based surface modification strategies.

### 2.2 Laser‐Induced Vapor Nanobubble Formation

Following surface characterization, the ability of the surface‐modified AuNPs to generate VNBs under pulsed laser irradiation was investigated, as this is preferred for efficient photoporation of cells [42]. Under dark‐field microscopy, the formation of VNBs was observed as short‐lived, bright scattering spots (Figure 2f) originating from the rapid thermal expansion and subsequent collapse of vapor bubbles around the AuNPs. To gain deeper insight into the VNB dynamics, high‐speed imaging was performed using a high‐frame‐rate sCMOS camera operating at 1000 frames per second (FPS), facilitating the visualization of the transient VNB lifecycle (Figure S2a,b). In Supporting Video S1, the process of VNB formation and collapse is presented at a 1000‐fold slower speed: VNBs are triggered immediately upon laser irradiation and complete their growth‐collapse cycle almost instantaneously; typically, VNBs appear and disappear within a single millisecond (Figure 2f).

To determine the laser fluence threshold for VNB formation, the number of VNBs was quantified across a range of increasing laser fluences (Figure 2g). The resulting data were fitted using a Boltzmann sigmoidal function to extract the threshold value, defined as the fluence at which 90% of the irradiated AuNPs generate VNBs [42, 43]. This analysis revealed a VNB threshold of 1.0 J cm^−2^, which was used as the reference fluence in subsequent photoporation experiments.

### 2.3 Intracellular Delivery of FITC‐Dextran

Next, we evaluated the potential of PBA‐functionalized AuNPs as photosensitizers for the intracellular delivery of macromolecules via photoporation. As a proof of concept, HeLa cells were selected due to their widespread use in photoporation research [44, 45]. FITC‐dextran with a molecular weight of 500 kDa (FD500) was employed as a model macromolecule because of its comparable size to eGFP‐mRNA (353 kDa, 996 nucleotides) [46]. To optimize delivery conditions, we tested a range of laser fluences and AuNP concentrations to identify combinations that maximize delivery efficiency while minimizing cytotoxicity. After establishing efficient delivery in HeLa cells, we extended the investigation to Jurkat cells, a commonly used suspension cell line that models primary human T cells [47]. Experimental details on the photoporation of the HeLa and Jurkat are described in the Materials and Methods section.

**FIGURE 3:**
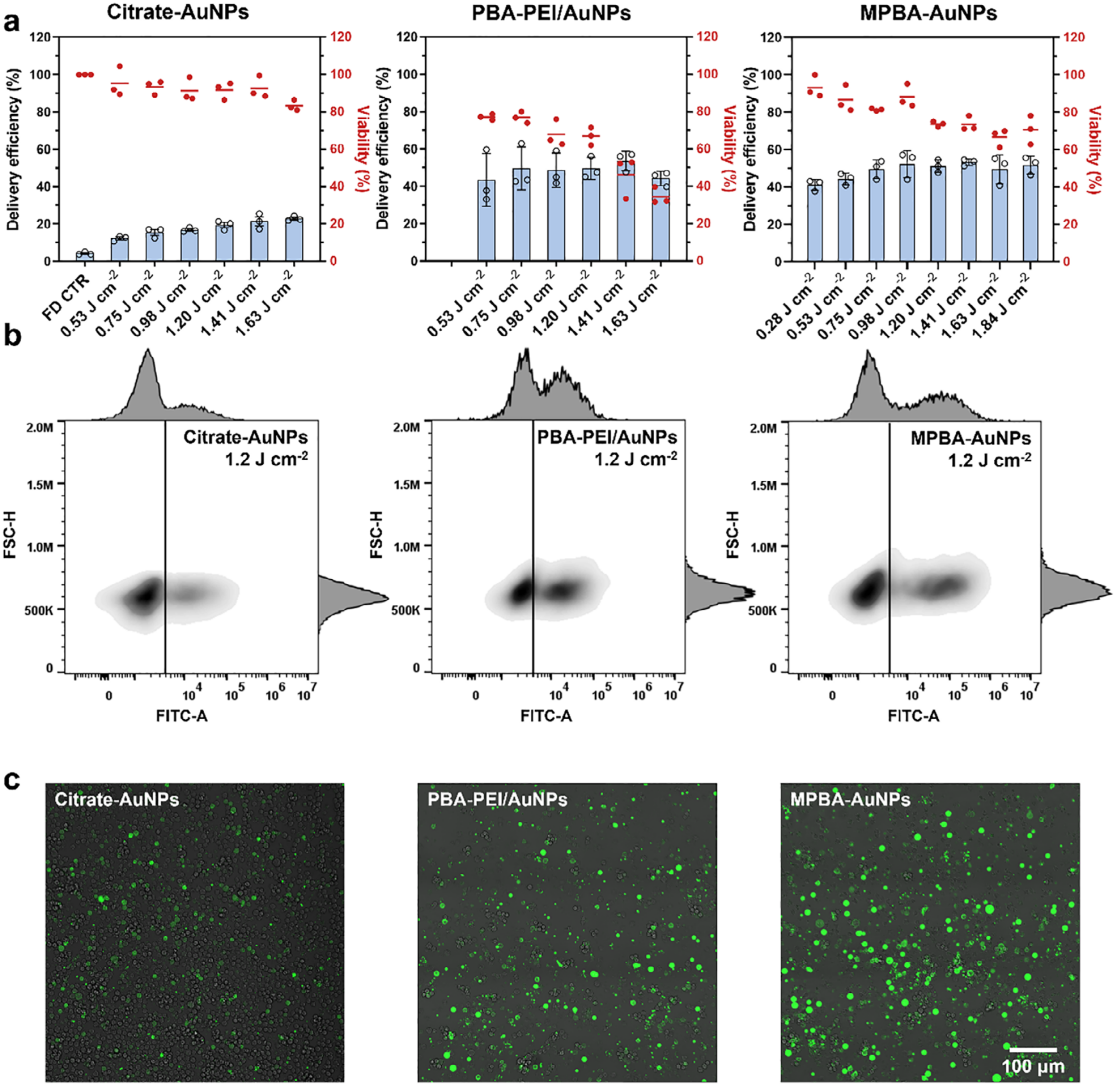
FD500 delivery in Jurkat cells via photoporation using AuNPs as photosensitizer. (a) Comparative analysis of delivery efficiency (i.e., the percentage of FD500‐positive cells; left Y‐axis) and cell viability (right Y‐axis) for citrate‐AuNPs, PBA‐PEI/AuNPs, and MPBA‐AuNPs across various laser fluences. The concentration of AuNPs in all experiments was fixed at 100 × 10^7^ NPs mL^−1^. (b) Representative flow cytometry density plots and histograms showing the population of FD500‐positive Jurkat cells following photoporation mediated by citrate‐AuNPs, PBA‐PEI/AuNPs, and MPBA‐AuNPs. All samples were treated at an AuNP concentration of 100 × 10^7^ NPs mL^−1^ and a laser fluence of 1.2 J cm^−2^. (c) Confocal fluorescence microscopy images of Jurkat cells after intracellular delivery of FD500 (green) using citrate‐AuNPs, PBA‐PEI/AuNPs, and MPBA‐AuNPs. Scale bar is 100 µm.

We first investigated unmodified citrate‐AuNPs under laser fluences ranging from 0.53 to 1.63 J cm^−2^ (Figure 3a, **left panel**). As the fluence increased, both delivery efficiency (percentage of FD500‐positive cells) and relative mean fluorescence intensity (rMFI; Figure S3) of the cells improved. At 1.63 J cm^−2^, citrate‐AuNPs achieved a delivery efficiency of ∼ 23% and an rMFI of ∼ 35, while maintaining a high cell viability of 83%. A reason why in only 23% of the cells FD500 could be delivered is, highly likely, the strong negative surface charge of citrate‐AuNPs which is repelled by negatively charged cell membranes, resulting in poor cell surface attachment of the AuNPs and thus limited photoporation efficacy.

In contrast, PBA‐PEI/AuNPs significantly enhanced FD500 delivery (Figure 3a, **middle panel**). At 1.2 J cm^−2^, PBA‐PEI/AuNPs resulted in ∼ 54% delivery efficiency, an rMFI of 30, and a delivery yield of 33% (Figure S4). However, the strong positive charge of PEI induced considerable cytotoxicity, indicating a trade‐off between sufficient membrane permeabilization and cell health.

MPBA‐functionalized AuNPs offered “a more balanced” profile. At the same laser fluence (1.2 J cm^−2^), MPBA‐AuNPs achieved ∼ 51% delivery efficiency (Figure 3a, **right panel**) and a much higher rMFI of 83 resulting in a delivery yield of ∼38% (Figure S5), with only moderate impact on cell viability. This suggests that MPBA allows sufficient MPBA functionalization promoted cell‐surface association, consistent with PBA‐mediated glycan interactions, however without the severe toxicity as seen with strongly cationic coatings.

Figure 3b presents representative flow cytometry data obtained on photoporated Jurkat cells (delivery of FD500) using citrate‐AuNPs (left), PBA‐PEI/AuNPs (middle), and MPBA‐AuNPs (right). Corresponding confocal fluorescence images of the Jurkat cells loaded with FD500 by photoporation are shown in Figure 3c. To visualize the influence of laser fluence and AuNP concentration, a 3D colormap response (delivery yield) surface was constructed (Figure S3c). Optimal photoporation conditions derived from these maps were as follows: 264 × 10^7^ NPs mL^−1^ and 1.2 J cm^−2^ for citrate‐AuNPs; 50 × 10^7^ NPs mL^−1^ and 1.0 J cm^−2^ for PBA‐PEI/AuNPs; and 100 × 10^7^ NPs mL^−1^ and 1.2 J cm^−2^ for MPBA‐AuNPs.

Notably, both PBA‐PEI and MPBA modification of the surface of the AuNPs significantly reduced the AuNP dose required to achieve efficient delivery. The cell membrane disruption by PEI itself (due to its cationic charges) likely explains that for PBA‐PEI/AuNPs a lower laser fluence is sufficient for optimal photoporation, though it comes at the cost of higher cytotoxicity. In contrast, MPBA‐AuNPs turned out to be an attractive photosensitizer as it allows efficient delivery of FD500 in cells while being relatively safe to the cells.

To further investigate the impact of MPBA surface modification on the “photoporation performance” of the AuNPs, we varied the MPBA concentration (as used in the coating process) while keeping the AuNP concentration at 100 × 10^7^ NPs mL^−1^ and using a fixed laser fluence of 1.2 J cm^−2^. As shown in Figure S5a, the delivery efficiency gradually increased up to ∼ 50% and levelled off above 1 µm of MPBA while cytotoxicity further increased. We then explored the effect of MPBA‐AuNPs dose (i.e., the concentration of AuNPs used in the photoporation experiments) at the optimal 1.0 µm MBA concentration (see above) and at a fixed laser fluence of 1.2 J cm^−2^. We tested MPBA‐AuNPs concentrations between 25 and 200 × 10^7^ NPs mL^−1^. As shown in Figure S5b, delivery efficiency increased from 26% to 55% with increasing MPBA‐AuNP concentration. However, this came at the cost of reduced viability, which dropped from 98% at the lowest dose to 51% at the highest MPBA‐AuNP concentration.

These findings confirm that both the MPBA concentration (as used in the coating process) and the MPBA‐AuNPs dose must be carefully balanced to maximize FD500 delivery into the cells while minimizing cytotoxicity. The optimal conditions identified in this study were: MPBA concentration at 1.0 µm, MPBA‐AuNP dose at 100 × 10^7^ NPs mL^−1^, and laser fluence at 1.2 J cm^−2^.

### 2.4 Intracellular Delivery of eGFP‐mRNA

Building upon the successful intracellular delivery of FITC‐dextran, next we studied whether MPBA‐AuNPs were suitable for photoporation mediated delivery of mRNA encoding enhanced green fluorescent protein (eGFP‐mRNA) in Jurkat cells. A fixed volume of 25 µL eGFP‐mRNA solution at a concentration of 0.1 µg µL^−1^ was added to each well. Photoporation was then performed using MPBA‐functionalized gold nanoparticles at different concentrations, under a laser fluence of 1.2 J cm^−2^.

**FIGURE 4:**
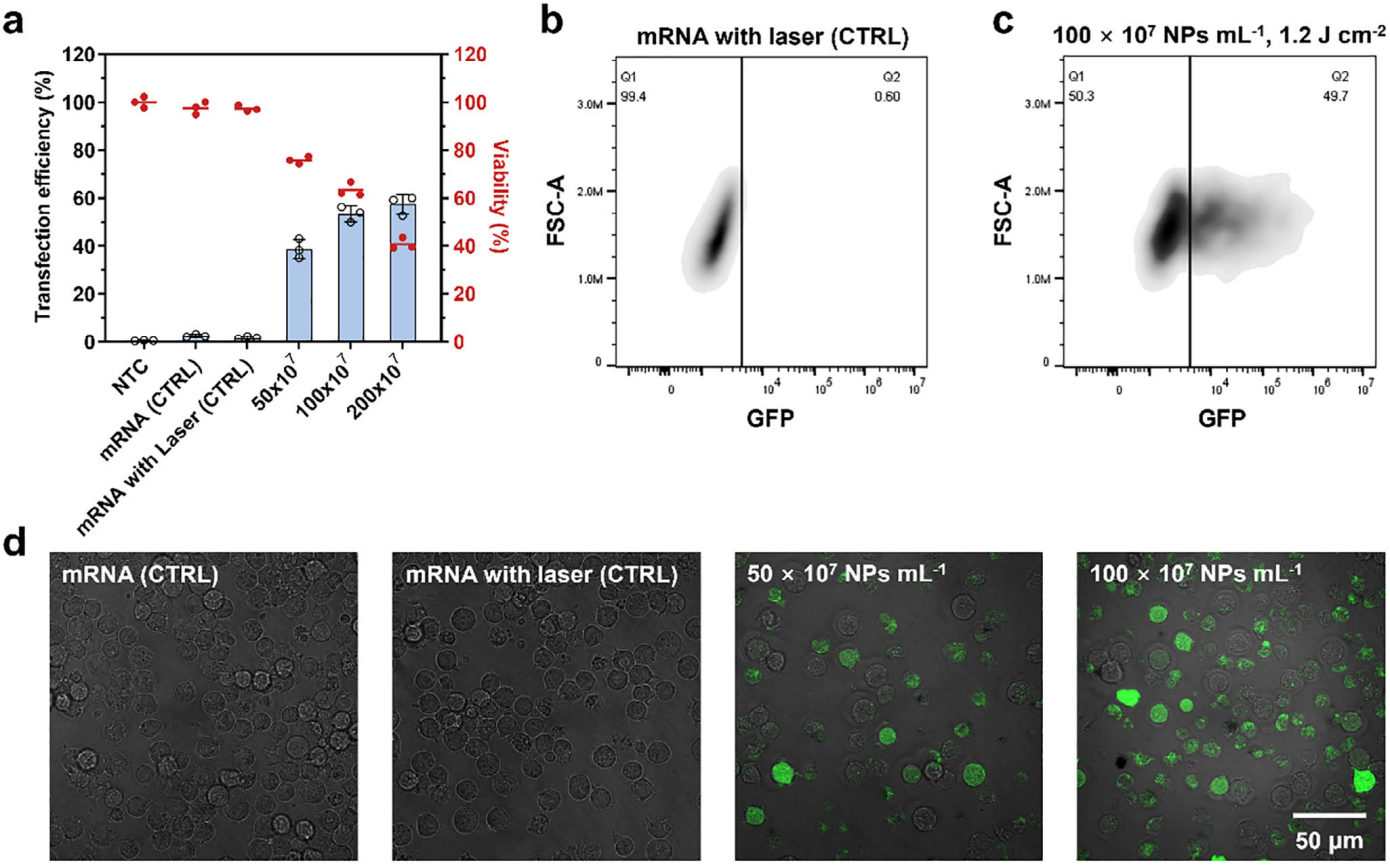
Optimization of photoporation mediated eGFP‐mRNA delivery into Jurkat cells using MPBA‐functionalized AuNPs. (a) Transfection efficiency (i.e., percentage of cells expressing GFP; left Y‐axis) and cell viability (right Y‐axis) of Jurkat cells treated with MPBA‐AuNPs at different dose (laser fluence: 1.2 J cm^−2^). (b,c) Representative flow cytometry plots showing eGFP expression in Jurkat cells for the following groups: (b) eGFP‐mRNA with laser treatment (control) and (c) eGFP‐mRNA with MPBA‐AuNPs and laser treatment. (d) Confocal fluorescence images of Jurkat cells showing eGFP expression in (from left to right) the eGFP‐mRNA only group (control), the eGFP‐mRNA with laser group (control), the eGFP‐mRNA with MPBA‐AuNPs and laser group (concentration of MPBA‐AuNPs was 50 × 10^7^ NPs mL^−1^ and 100 × 10^7^ NPs mL^−1^, respectively). Scale bar is 50 µm. Data are presented as mean ± SD (*N* = 3, *n* = 3).

As Figure 4 shows, in the absence of AuNPs, cells exposed to mRNA and laser irradiated were almost not transfected. In contrast, cells treated with 50 × 10^7^ NPs mL^−1^ MPBA‐AuNPs achieved an average transfection efficiency (i.e., percentage of cells expressing eGFP) of ∼39%, with a rMFI of 190 and a transfection yield of ∼29% (Figure S6). Increasing the MPBA‐AuNP concentration further enhanced transfection performance though cell viability further dropped (Figure 4a). Flow cytometry density plots (Figure 4b,c) clearly demonstrate the critical role of the MPBA‐AuNPs in enabling mRNA delivery through photoporation. To visually confirm successful transfection, Jurkat cells were further analyzed using confocal fluorescence microscopy. Representative images (Figure 4d) show merged bright‐field and green fluorescence channels which confirmed effective and dose‐dependent transfection enabled by MPBA‐AuNPs.

### 2.5 Detaching AuNPs from the Cell Surface

Phenylboronic acid is known for its ability to form reversible covalent boronate ester bonds with *cis‐*diol‐containing molecules. These dynamic interactions can be competitively disrupted by small molecules rich in *cis*‐diol groups when present at sufficiently high concentrations [48]. We explored the potential of adenosine triphosphate (ATP) to trigger detachment of PBA‐functionalized AuNPs from the cell surface. ATP was selected because its *cis‐*diol groups can competitively interact with PBA moieties. ATP is normally present at relatively low concentrations in the extracellular environment (<0.4 mm), whereas its intracellular concentration is substantially higher, typically in the range of 1–10 mm [49, 50]. In the present study, 4 mm ATP was applied as a short‐term processing condition to promote competitive displacement of the boronate ester interactions and maximize AuNP detachment. Importantly, exposing Jurkat cells to 4 mm ATP for 5 min maintained approximately 91% cell recovery after 24 h, without a marked increase in cell‐normalized Caspase‐3/7 activity (Figure S10). This concentration should therefore be regarded as a supraphysiological extracellular processing concentration rather than as a simulation of the physiological extracellular environment.

**FIGURE 5:**
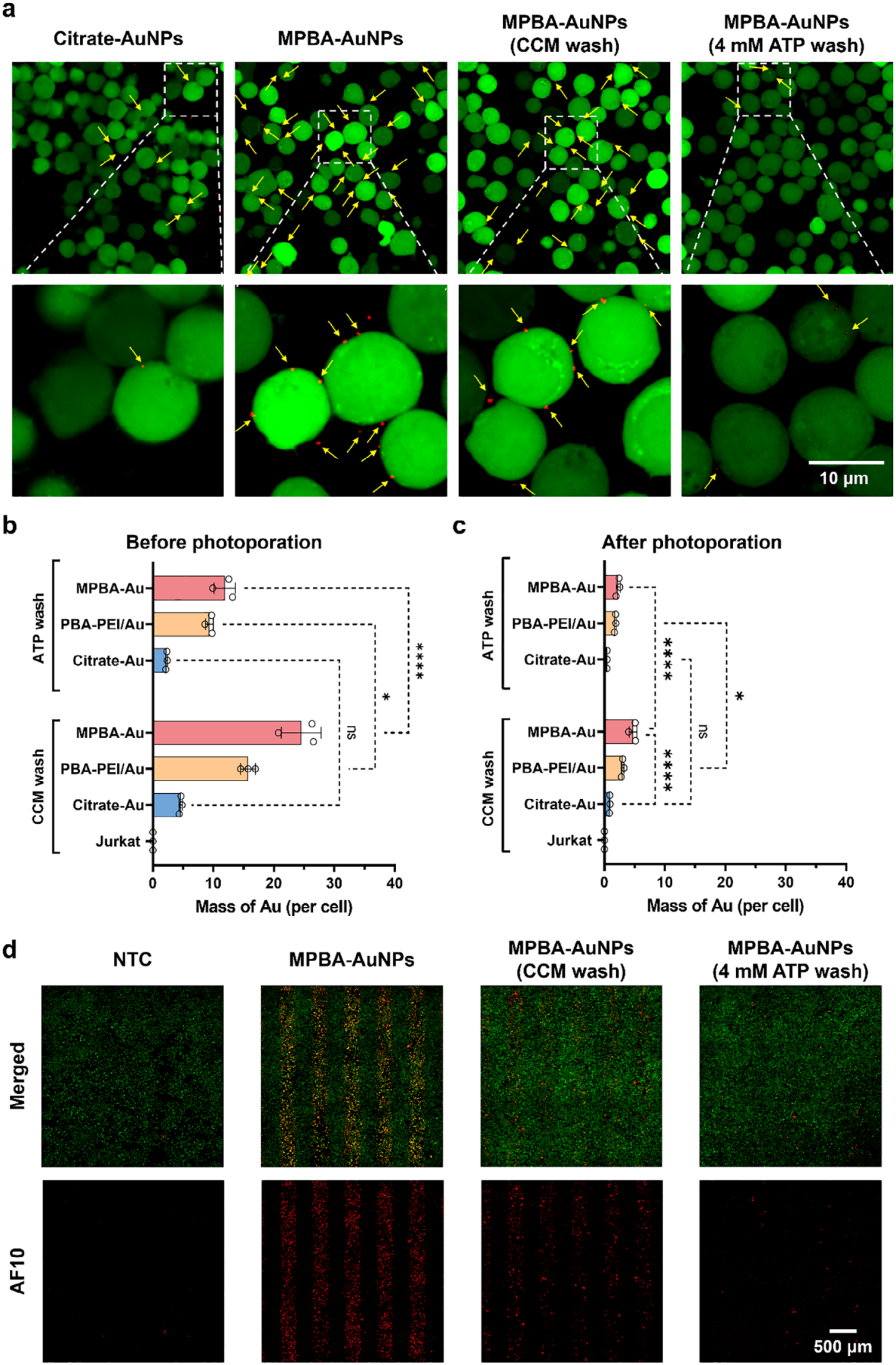
Detachment of AuNPs from the cell surface. Confocal images showing the attachment/detachment of AuNPs to/from the cell surface; AuNPs (red dots) could be detected as they strongly scatter light. (a) Citrate‐AuNPs (served as a non‐boronic reference control) and MPBA‐AuNPs on the surface of viable Jurkat cells (stained in green with calcein‐AM), before and after washing with cell culture medium (CCM) or CCM supplemented with 4 mM ATP. Scale bar is 10 µm. (b) Total cell‐associated gold mass per viable cell, as measured by inductively coupled plasma mass spectrometry (ICP‐MS), demonstrating AuNP attachment and ATP‐induced detachment; cells were not laser irradiated. (c) Total cell‐associated gold mass per viable cell after photoporation, as measured by ICP‐MS. Data are presented as mean ± SD of three independent experiments (*N* = 3). ns: *p* ≥ 0.05, ^*^: *p* < 0.05, ^**^: *p* < 0.01, ^***^: *p* < 0.001, ^****^: *p* < 0.0001. (d) HeLa cells were seeded and allowed to adhere until reaching 90% confluency. MPBA‐AuNPs were added to the cells (incubation of 10 min). Next, cells were washed and red Alexa Fluor 488‐labeled dextran (10 000 MW, AF10, 0.1 mg mL^−1^) was added. **First row** (outmost left): in HeLa cells which were not exposed to MPBA‐AuNPs and which did not receive laser treatment (no‐treatment control group; NTC) there was no AF1010. **Second row**: photoporation of HeLa cells having MPBA‐AuNPs at their surface (i.e., cells which were not yet washed) clearly delivered AF1010 in the cells. **Third row**: washing the cells with cell culture medium (CCM wash) and subsequent laser irradiation still resulted in the presence of AF10 in the cells**. Fourth row** (outmost right): washing the cells with CCM supplemented with 4 mM ATP showed a near‐complete absence of intracellular AF10. Scale bar is 500 µm.

To visualize attachment/detachment of AuNPs to/from the surface of cells, we employed the scatter detection mode on a confocal microscope. As explained in the experimental section, cell suspensions were incubated with respectively citrate‐AuNPs or MPBA‐AuNPs at equal concentrations. After gentle agitation of the 96‐well plate, followed by a 5 min equilibration period, the supernatant containing unbound AuNPs was removed by aspiration and, subsequently, fresh cell culture medium was added. As Figure 5a shows, confocal imaging revealed only sparse surface binding for citrate‐AuNPs (as expected, considering their negative charge). In contrast, a significantly higher number of MPBA‐modified AuNPs was observed at the cell surface.

Interestingly, as Figure 5a shows as well, while washing with cell culture medium (CCM) proved insufficient to remove the majority of MPBA‐AuNPs, a single wash with CCM supplemented with 4 mM ATP successfully triggered the detachment of a substantial number of MPBA‐AuNPs. We consider this detachment is driven by the competitive binding of the *cis‐*diol groups in ATP to the PBA moieties, which displaces the boronate ester bonds between AuNPs and cell‐surface glycans. Experiments performed on adherent HeLa cells yielded similar results, as shown in Figure S7.

To quantify the attachment and detachment of the AuNPs, inductively coupled plasma mass spectrometry (ICP‐MS) analysis was performed on Jurkat cells. Detachment of the AuNPs from the surface of the cells was studied respectively before (Figure 5b) and after the photoporation step (Figure 5c). For a more intuitive interpretation, the measured total gold mass was also converted into the average number of nanoparticles per cell, based on a theoretical mass of 5.17 fg for a single 80 nm spherical AuNP. These mass‐equivalent values should not be interpreted as physical counts of intact nanoparticles.

Analysis of cells which were not laser irradiated (thus not photoporated; Figure 5b) revealed that after washing with CCM, the average cell‐associated gold mass was 4.61 fg for citrate‐AuNPs, 15.74 fg for PBA‐PEI/AuNPs, and 24.53 fg for MPBA‐AuNPs per viable cell. These values correspond to 0.89, 3.04, and 4.74 theoretical 80 nm AuNP mass equivalents per viable cell, respectively. These data confirm that PBA functionalization significantly enhances the attachment of AuNPs to Jurkat cell membranes. Washing with CCM containing 4 mM ATP decreased the gold mass of MPBA‐AuNP treated cells from 24.53 to 11.88 fg Au per viable cell, corresponding to a decrease from 4.74 to 2.30 AuNP mass equivalents. A smaller decrease was observed for the unmodified citrate‐AuNPs. This selective removal highlights the advantage of the dynamic boronate ester bonds, which are effectively displaced by the competitive binding of ATP's *cis‐*diol groups.

Figure 5c shows the residual gold levels following laser‐irradiation (photoporation) of the cells. First, the overall amount of residual AuNPs was lower (when compared to the non‐irradiated cells in Figure 5b). This reduction is, most likely, primarily due to a portion of the AuNPs undergoing laser‐induced fragmentation, facilitating the removal of the AuNPs during the washing steps. Interestingly, washing with ATP further reduced the gold mass of MPBA‐AuNP treated cells to 2.30 fg Au per viable cell. This corresponds to 0.44 theoretical 80 nm AuNP mass equivalents per viable cell and is below the theoretical mass of one spherical 80 nm AuNP. Our data thus suggest that the combined use of phenylboronic acid based chemistry and ATP allows an almost complete removal of the gold photosensitizer.

To further confirm detachment of the AuNPs from the cells we verified the absence of intracellular delivery of Alexa Fluor 488‐labeled dextran (10 000 MW, AF10) into cells upon subsequent laser irradiation. If AuNPs are effectively removed, photoporation should no longer be effective and we expect little to no AF10 to be delivered into the cells. For laser treatment, the well‐plates with the cells were placed on an electronic motorized translation stage which facilitated automated laser scanning across each well; the laser beam followed a serpentine pattern which allowed to photoporate cells in specific regions of the cell culture (as demonstrated in Figure S8). As Figure 5d shows, and most importantly, washing the cells with CCM supplemented with 4 mM ATP showed a near‐complete absence of intracellular AF10 which demonstrates that the vast majority of MPBA‐AuNPs was successfully detached from the cells.

To examine cellular recovery beyond conventional short‐term viability measurements, we monitored cell proliferation for 7 days after treatment. Jurkat cells continued to proliferate in all groups, although the two photoporation groups showed moderately lower cumulative cell yields than untreated cells on day 7 (Figure S11). AD‐MSCs likewise maintained sustained proliferation and day‐7 metabolic activity following the complete photoporation and ATP‐mediated detachment procedure, despite an increased cell‐normalized Caspase‐3/7 signal at 24 h (Figure S12).

## 3 Conclusions

Efficient and safe intracellular delivery of macromolecules remains a major challenge in cell engineering. While photoporation is a highly attractive method for cytosolic delivery of (macro)molecules, photosensitizers remaining at/in the photoporated cells may pose (long‐term) safety concerns, especially when the cells are engineered for therapeutic use. We addressed this concern and introduced the concept of “detachable photosensitizers” using AuNPs as photosensitive nanoparticles.

By leveraging the reversible nature of boronate ester bonds, we demonstrated that phenylboronic acid (PBA)‐modified AuNPs can be attached to cell membranes, enabling the intracellular delivery of high‐molecular‐weight dextrans and mRNA upon pulsed laser irradiation. Subsequently, the AuNPs can be detached from the cells on demand through ATP‐triggered competitive binding. We confirmed that the detachment process markedly reduced the average cell‐associated gold mass per viable cell to below the theoretical mass of a single intact 80 nm AuNP, minimizing foreign material residues in the cells.

Although the present study primarily used Jurkat cells as a model suspension T‐cell line, complementary experiments in adipose‐derived mesenchymal stromal cells (AD‐MSCs) further supported the applicability of the workflow to an adherent, clinically relevant stromal cell type. The detachable photosensitizer strategy may, in principle, be further extended to primary immune cells and other clinically relevant cells displaying accessible *cis*‐diol‐containing glycans and glycoconjugates on their plasma membranes. Such an extension could be particularly relevant to the ex vivo engineering of primary T cells, natural killer cells, and hematopoietic stem and progenitor cells, for which minimizing residual foreign materials is important. Nevertheless, differences in cell‐surface glycosylation, nanoparticle‐binding capacity, membrane sensitivity, and cellular responses to laser irradiation may affect both photoporation and ATP‐triggered AuNP detachment. The different post‐treatment recovery profiles observed for Jurkat cells and AD‐MSCs may also partly reflect the use of identical AuNP concentrations for standardized comparison rather than cell‐type‐specific optimization. Future studies should therefore optimize the attachment, photoporation, and detachment conditions for each primary cell type and assess not only delivery efficiency and viability, but also phenotype, proliferative capacity, and cell‐specific functions following treatment.

Overall, the “attach‐and‐detach” strategy introduced here offers a straightforward approach to reducing the residual photosensitizer burden after photoporation and may facilitate safer and more controlled manufacturing of engineered cells for cell therapy.

## 4 Experimental Section

### 4.1 Materials

80 nm citrate‐AuNPs were provided by Trince BV (Belgium). 4‐carboxyphenylboronic acid, polyethylenimine (PEI, Branched, average Mw ∼25 000, Cat. No. 40 872‐7), N‐(3‐Dimethylaminopropyl)‐N′‐ethylcarbodiimide hydrochloride (EDC·HCl, ≥98.0%, MW 191.70, CAS No. 25952‐53‐8) and adenosine 5′‐triphosphate (ATP) disodium salt hydrate (crystalline, ≥97%) were purchased from Sigma‐Aldrich (Belgium). 4‐mercaptophenylboronic acid (MPBA) was purchased from TCI Europe N.V. Ethanol (abs. 100% a.r.) and Dimethyl sulfoxide (DMSO, a.r., 99.9+%) were purchased from Chem‐Lab NV (Zedelgem, Belgium). Dulbecco's Modified Eagle's Medium/Nutrient Mixture F‐12 (DMEM/F‐12), Roswell Park Memorial Insitute (RPMI) 1640 medium, penicillin/streptomycin solution (100 IU mL^−1^ penicillin, 100 µg mL^−1^ streptomycin), L‐glutamine (2 mm) and 0.25% trypsin‐EDTA Gibco and collagen I, rat tail (Cat No. A1048301) and 0.4% trypan blue solution were purchased from Gibco‐Life Technologies (USA). Fetal bovine serum (FBS) was from Hyclone (GE Healthcare, Belgium). Nuclease‐free water (Cat No. AM9937), Dulbecco's phosphate‐buffered saline without CA^2+^/Mg^2+^ (DPBS‐), bovine serum albumin (BSA), and sodium azide (NaN_3_) were obtained from Sigma‐Aldrich (USA). TO‐PRO‐3 Iodide (Cat No. T3605), Calcein‐AM (Cat No. C3099) and Alexa Fluor 488‐labeled dextran (10 000 MW, anionic, fixable) were obtained from Invitrogen (USA). The CellTiter‐Glo Luminescent Cell Viability Assay (Cat No. G7571) was obtained from Promega (Belgium).

### 4.2 Cell Lines and Culture Conditions

Human cervical epithelial adenocarcinoma HeLa cells and T lymphoblast Jurkat E6‐1 cells were obtained from the American Type Culture Collection (ATCC, USA). HeLa cells were cultured in DMEM/F‐12 medium. Jurkat cells were cultured in RPMI 1640 medium. All culture media were supplemented with 10% FBS, 2 mm L‐glutamine and 100 U mL^−1^ penicillin/streptomycin (i.e., complete cell culture medium). All cell lines were maintained in a humidified atmosphere containing 5% CO_2_ at 37°C and culture medium was renewed every other day. To ensure consistent experimental conditions and phenotypic stability, all experiments were conducted using cells with a passage number below 20. Cell lines are regularly tested and were found negative for mycoplasma.

### 4.3 Synthesis of PBA‐PEI

The functionalized polymer, phenylboronic acid‐grafted polyethylenimine (PBA‐PEI), was synthesized via a carbodiimide‐mediated amidation reaction, which facilitates the formation of stable covalent amide linkages between the carboxylic acid groups of the phenylboronic acid ligand and the amine groups of PEI (Figure S1) [50]. The reaction principle involves a two‐step sequence where the carboxyl groups of 4‐carboxyphenylboronic acid (CPBA) first react with EDC·HCl (1‐ethyl‐3‐(3‐dimethylaminopropyl)carbodiimide hydrochloride) to generate a highly reactive *O*‐acylisourea intermediate. This intermediate is subsequently subjected to nucleophilic attack by the primary and secondary amine groups of the branched PEI, resulting in the formation of a permanent amide bond (–CO–NH–) and the release of a water‐soluble urea byproduct. Maintaining a pH of approximately 5.5 during the process is essential to optimize the balance between the stability of the activated intermediate and the nucleophilicity of the PEI amines, ensuring a high grafting density of phenylboronic acid moieties onto PEI.

PBA‐PEI was synthesized as follows. 200 mg of CPBA was dissolved in 40 mL of a mixture of ethanol and deionized water (1:1, v/v). To this solution, 800 mg of EDC·HCl was added and allowed to react at room temperature for 1 h to fully activate the carboxyl groups of the PBA. Simultaneously, 400 mg of branched PEI was dissolved in 20 mL of the same solvent mixture, with the pH carefully adjusted to 5.5. Following the activation period, the CPBA solution was added dropwise into the PEI solution under constant stirring to prevent non‐specific aggregation or precipitation. The mixture was stirred at room temperature for 24 h to ensure complete conjugation, after which the crude product was transferred to a dialysis kit with a molecular weight cut‐off (MWCO) of 12–14 kDa (Pur‐A‐Lyzer Mega Dialysis Kit, Sigma‐Aldrich). The solution was dialyzed against Milli‐Q water for three days with frequent water changes to remove unreacted CPBA, EDC byproducts, and residual solvents, yielding the purified PBA‐PEI copolymer ready for subsequent surface modification of the gold nanoparticles. Finally, the PBA content was determined by measuring and comparing the characteristic absorbance peak at 268 nm of the purified PBA‐PEI solution against a standard PBA calibration curve using a NanoDrop 2000 spectrophotometer (Thermo Scientific).

### 4.4 Surface Functionalization of AuNPs via PBA‐PEI or MPBA

Citrate‐AuNPs were mixed with solutions of PBA‐PEI or MPBA and incubated overnight at room temperature. The mixture was subjected to continuous agitation at 500 rpm using a digital shaker (IKA MS 3) to facilitate surface functionalization of the AuNPs via either electrostatic interaction (for PBA‐PEI) or Au‐S covalent bonding (for MPBA).

Surface functionalization via Au–S covalent bonding. For the preparation of MPBA‐functionalized AuNPs, MPBA was first dissolved in dimethyl sulfoxide (DMSO) to create concentrated stock solutions. Subsequently, 10 µL of MPBA stock solution was added to 990 µL of the citrate‐AuNPs suspension. The AuNP concentration equaled to 5 × 10^10^ nanoparticles per milliliter (NPs mL^−1^), as determined using a Zetasizer Pro (Malvern). The MPBA concentration in the stock solutions was calculated to achieve MPBA concentrations of respectively 0.25, 0.5, 1.0, and 2.0 µm after addition to the AuNP dispersion.

Surface functionalization via electrostatic attraction. 990 µL of a citrate‐AuNP dispersion (5 × 10^10^ NPs mL^−1^) was mixed with 10 µL of a PBA‐PEI solution. The mixture was shaken overnight to ensure sufficient time for the electrostatic binding of the PBA‐PEI to the AuNPs.

Removal of unreacted ligands. To remove unreacted agents (MPBA / PBA‐PEI), the mixtures above were centrifuged at 4000 rcf for 10 min, followed by the careful aspiration and disposal of the supernatant. The resulting pellets of PBA‐PEI/AuNPs and MPBA‐AuNPs were then resuspended in nuclease‐free water for subsequent use.

The hydrodynamic size and zeta potential of the AuNPs were measured by a Zetasizer Nano‐ZS90 (Malvern), UV–vis absorption spectra were taken by a NanoDrop 2000c (Thermo Scientific) while morphological characteristics were measured via Transmission electron microscopy (TEM, JEM1010, JEOL).

### 4.5 Determination of the Vapor Nanobubble Generation Threshold for AuNPs

The formation of vapor nanobubbles (VNBs) from AuNPs was studied using a previously reported (in‐house developed) setup [24, 25]. The system includes a pulsed laser source (Opolette HE 355 LD) with a pulse duration of approximately 7 ns, a repetition rate of 20 Hz, and a beam diameter of ∼ 200 µm. The excitation wavelength was set to 532 nm. The stocks of AuNPs were first diluted in ddH_2_O to a concentration of 5 × 10^10^ NPs mL^−1^. Samples were then transferred into 50 mm γ‐irradiated glass bottom dishes (MatTek Corporation) and irradiated with laser pulses at varying laser fluence. VNBs were visualized via dark‐field microscopy using a dark‐field condenser, capitalizing on the strong light scattering properties of the nanobubbles. An EMCCD camera (Cascade II: 512) was synchronized with the laser pulses through an electronic pulse generator (BNC575), enabling the acquisition of images before, during, and after laser exposure. Laser pulse energy was monitored in real time using an energy meter (J‐25MB‐HE&LE, EnergyMax‐USB/RS sensors, Coherent), and laser fluence was calculated as the average energy per pulse divided by the beam area (J cm^−2^).

To facilitate the identification and quantification of individual VNBs against the background, frame‐by‐frame image analysis was performed using ImageJ (FIJI, https://fiji.sc/), allowing precise visualization of VNB formation and collapse dynamics. As our group reported before [25, 51], by counting the number of VNBs within the laser irradiation area for increasing laser fluence, we could determine the VNB threshold, defined as the laser fluence which resulted in 90% of the maximal number of nanobubbles which could be detected in the irradatiated zone (details can be found in the **Note** **S1.1**).

### 4.6 Intracellular Delivery of FITC‐Dextran by Photoporation

FITC‐dextran (500 kDa; FD500, Sigma–Aldrich) was initially used as a model macromolecule to optimize photoporation parameters for intracellular delivery. Experiments were conducted with both HeLa and Jurkat cells. As HeLa cells are adherent cells, they were seeded in flat‐bottom 96‐well plates at a density of 1 × 10^4^ cells per well 24 h prior to the experiment. Non‐adherent Jurkat cells were seeded into U‐bottom 96‐well plates at a density of 2.5 × 10^5^ cells per well. Subsequently, surface‐modified AuNP dispersions were prepared in Opti‐MEM and added to the cells in the wells; the AuNP concentration was varied: 25, 50, 100, and 200 × 10^7^ NPs mL^−1^. Following (i) gentle agitation (100 rpm, 5 min) of the 96‐well plate using an orbital shaker and (ii) a 5 min equilibration period at room temperature, unbound AuNPs were removed as follows: the plate was first centrifuged (300 rcf, 5 min) using a microplate‐compatible centrifuge (Eppendorf), followed by the careful aspiration of the supernatant containing unbound AuNPs.

Next, 50 µL of FD500 (1 mg mL^−1^ concentration) was added to the cells in the wells, having AuNPs at their surface. For Jurkat cells, the resulting cell suspensions were carefully transferred into the wells of a flat‐bottom 96‐well plate, this to allow irradiation with light (photoporation). The plate was briefly centrifuged (∼ 10 s, 0–1000 rpm) to ensure the cells formed a uniform sediment at the bottom of the wells. Photoporation was then performed at 532 nm (nanosecond pulsed laser) using the commercially available benchtop photoporation device (LumiPore, Trince, Belgium). Following laser treatment, the cell suspensions were then transferred into the wells of a U‐bottom 96‐well plate (to allow centrifuagtion), washed one time (centrifugation 300 rcf, 5 min) with cell culture medium supplemented with 4 mm ATP, this to remove extracellular FD500 and detach the AuNPs from the cells. Finally, the cell pellets were resuspended in fresh cell culture medium and incubated at 37°C and 5% CO_2_.

To measure the ‘delivery efficiency’, i.e., the percentage of FD500‐positive cells, photoporated Jurkat cells in the wells were centrifuged (one time, 300 rcf, 5 min) and resuspended in flow buffer (DPBS‐, 1% BSA, 0.1% sodium azide) with 1 µm TO‐PRO‐3 iodide (Invitrogen, Belgium) as cell viability dye. Flow cytometry was performed using a CytoFLEX flow cytometer (Beckman Coulter) and a minimum of 2 × 10^4^ cells were analyzed per sample. FITC and TO‐PRO‐3/APC were excited with 488 and 640 nm lasers and detected with 525/50 and 655–730 nm filters, respectively. FlowJo software (Treestar Inc.) was used for data analysis. Experimental details are described in **Note** **S1.2** which also explains how the ‘delivery yield’ was calculated.

### 4.7 Intracellular Delivery of eGFP‐mRNA by Photoporation

The photoporation experiments to transfect the cells with eGFP‐mRNA followed a protocol similar to that described above for FD500 delivery, with specific modifications for nucleic acid handling. Briefly, Jurkat cells were harvested in FBS‐containing culture medium, transferred in a U‐bottom 96‐well plate (2.5 × 10^5^ cells per well) and washed three times via centrifugation (300 rcf, 5 min) with Opti‐MEM. Following the third wash, 50 µL of MPBA‐AuNPs suspension (at concentrations of 50, 100 or 200 × 10^7^ NPs mL^−1^) was added to the cells in the wells. Subsequently, the cells were gently agitated (100 rpm, 5 min) using an orbital shaker and allowed to equilibrate for 5 min at room temperature. Unbound AuNPs were then removed by centrifugation (300 rcf, 5 min) and aspiration of the supernatant to ensure only attached nanoparticles remained.

Next, 50 µL of eGFP‐mRNA solution (0.1 µg µL^−1^ in Opti‐MEM) was added to the cells in the wells, having AuNPs at their surface. To irradiate the cells with laser pulses (photoporation), the eGFP‐mRNA/cell suspensions were carefully transferred to a flat‐bottom 96‐well plate and briefly centrifuged (∼ 10 s, 0–1000 rpm) to ensure cells formed a uniform sediment at the bottom of the wells. Photoporation was then immediately performed using the nanosecond pulsed laser at specified laser fluences.

MPBA‐functionalized AuNPs were detached from the cells as follows. Following laser treatment, the cell suspensions were transferred into the wells of a U‐bottom 96‐well plate, then centrifuged (300 rcf, 5 min), and the supernatant was aspirated. Subsequently, 150 µL of RPMI 1640 medium supplemented with 4 mM ATP was added to the wells to promote competitive interaction with PBA moieties and facilitate AuNP detachment. Following the final washing with ATP, 200 µL of pre‐warmed complete cell culture medium was added to each well to facilitate cell recovery. The cells were then incubated at 37°C in a humidified 5% CO_2_ atmosphere for 24 h to allow for eGFP expression prior to downstream analysis via flow cytometry or confocal microscopy. The overall workflow of the photoporation experiments and subsequent post‐treatment of the cells is illustrated in Figure S9.

Three control groups were included in the experiments: (i) a ‘no‐treatment control’ (NTC) group: in which cells received 50 µL of Opti‐MEM without mRNA and without laser treatment; (ii) an ‘mRNA control’ (mRNA CTR) group: in which cells were incubated with 50 µL of mRNA (0.1 µg µL^−1^) but remained unirradiated; and (iii) an ‘mRNA with laser control’ group: in which cells were incubated with 50 µL of mRNA (0.1 µg µL^−1^) and laser irradiated though without the use of AuNPs.

### 4.8 Cell Health Evaluation

The CellTiter Glo luminescent cell viability assay (Promega, Belgium) was used according to the manufacturer's instructions to assess cell viability after photoporation. Briefly, cells in complete culture medium were supplemented with an equal volume of CellTiter Glo reagent and shaken on an orbital shaker (100 rpm) for 10 min at room temperature. Next, the cell lysates were transferred to an opaque 96‐well plate and the luminescent signal was measured using a GloMax microplate reader (Promega, Belgium) with a detection wavelength range of 350 to 650 nm. Cell viability was calculated relative to the non‐treated control.

### 4.9 Visualizing Attachment/Detachment of AuNPs by Confocal Miscroscopy

Cells were seeded in 96‐well glass‐bottom high‐content imaging plates (Corning). The use of such glass‐bottom plates with superior optical properties and lower auto‐fluorescence is essential for minimizing spherical aberration and light scattering during high‐magnification confocal acquisition. The procedures for the attachment and detachment of AuNPs to the cells in these wells were identical to those described in Sections 4.6 and 4.7. Cells were stained with Calcein‐AM (Invitrogen) according to the manufacturer's instructions, followed by the addition of fresh culture medium for subsequent imaging.

To visualize the AuNPs on the cell surface, imaging was performed using a Nikon A1plus confocal microscope mounted on a Ti2 inverted base (Nikon, Tokyo, Japan). A SR Plan Apo IR AC 60x water‐immersion objective (NA 1.27; refractive index 1.333) was used for all acquisitions. Dual‐channel imaging was implemented using high‐sensitivity GaAsP detectors. Live cells were visualized via green fluorescence from Calcein‐AM using a 487.7 nm laser line for excitation and a 525/50 nm emission filter. Simultaneously, the localization of AuNPs was monitored in scatter detection mode (pseudo‐colored red in merged images) by employing a 561.5 nm laser line. The optical pathway for scatter detection was configured using a BS 20/80 dichroic mirror with the emission filter set to “through”. The pinhole size was fixed at 37.04 µm for all channels. To comprehensively capture the AuNP distribution across the cell surface, Z‐stack imaging was conducted with a step size of 0.4 µm over 50 planes, covering a total vertical range of approximately 20 µm.

### 4.10 Quantifying the Number of AuNPs Bound Per Cell by ICP‐MS

To determine the number of AuNPs associated with Jurkat cells, inductively coupled plasma mass spectrometry (ICP‐MS) was performed. Jurkat cells were subjected to acid digestion in pre‐cleaned Teflon beakers (Savillex). Digestion was carried out using a mixture of 0.25 mL 14 m HNO_3_ and 0.75 mL 12 m HCl (1 mL of *aqua regia*) at 110°C for 18 h. Following digestion, samples were evaporated to dryness, redissolved in 1 mL of 10% aqua regia, and appropriately diluted (10‐ to 100‐fold) with 2% aqua regia prior to analysis.

Elemental quantification of Au was performed using an Agilent 7900 ICP‐MS instrument (Agilent Technologies) equipped with a MicroMist concentric nebulizer and a Scott‐type spray chamber. The instrument was operated in “no gas mode” with an RF power of 1550 W, a plasma gas flow rate of 15.0 L min^−1^, and a nebulizer gas flow rate of 1.10 L min^−1^. External calibration was established using Au standards ranging from 0 to 2 µg L^−1^, with ^195^Pt (1 µg L^−1^) monitored as an internal standard to correct for potential signal drift and matrix effects. The limits of detection and quantification for Au were determined to be 0.006 ng and 0.02 ng, respectively.

The total cell‐associated gold mass in each sample was quantified by ICP‐MS. Before ICP‐MS analysis, the number of viable cells in each sample was determined using a microscopy‐based cell counter after staining nonviable cells with 0.4% trypan blue. The measured gold mass was normalized to the number of viable cells and expressed as fg Au per viable cell. For intuitive comparison, the gold mass was also divided by the theoretical mass of one spherical 80 nm AuNP, approximately 5.17 fg, calculated using the bulk density of gold (19.3 g cm^−3^). The resulting values were expressed as 80 nm AuNP mass equivalents per viable cell. These mass‐equivalent values do not represent physical particle counts because ICP‐MS cannot distinguish among intact AuNPs, gold‐containing fragments, and dissolved gold species.

### 4.11 Statistical Analysis

Experiments were performed in biological triplicate (N = 3), with each biological replicate including at least three technical replicates. Unless otherwise stated, data are presented as the mean ± standard deviation (SD). Statistical analyses were performed using GraphPad Prism 9. Multiple conditions were compared using one‐way analysis of variance (ANOVA), followed by Tukey's multiple‐comparisons test.

## Author Contributions

**Tao Lu**: conceptualization, methodology, investigation, software, formal analysis, validation, visualization, Writing – original draft, Writing – review and editing. **Mina Nikolić**: methodology, data curation, formal analysis, validation, investigation, writing – review and editing. **Baihao Huang**: writing – review and editing, methodology, visualization, data curation. **Deep Punj**: methodology, software, data curation, validation. **Herlinde De Keersmaecker**: methodology, software, data curation, visualization. **Thibaut Van Acker**: methodology, validation, writing – review and editing, formal analysis, software. **Wenjia Xie**: methodology, investigation, validation, writing – review and editing, resources. **Florian Vanlauwe**: resources, methodology, investigation, writing – review and editing. **Phillip Blondeel**: resources, methodology, investigation, writing – review and editing. **Liesl De Graeve**: resources, methodology, investigation. **Félix Sauvage**: supervision, writing – review and editing, investigation, conceptualization. **Ranhua Xiong**: writing – review and editing, investigation, supervision, conceptualization. **Chaobo Huang**: conceptualization, investigation, writing – review and editing, supervision. **Frank Vanhaecke**: methodology, software, supervision, resources, writing – review and editing, conceptualization. **Kevin Braeckmans**: conceptualization, methodology, writing – review and editing, project administration, supervision, investigation. **Stefaan C. De Smedt**: writing – review and editing, writing – original draft, conceptualization, methodology, supervision, project administration.

## Funding

This work was supported by the China Scholarship Council (Grant No. 202208320061), National Natural Science Foundation of China (22275093, 22275094), and Natural Science Foundation of Jiangsu Province for Distinguished Young Scholars (BK20230008).

## Conflicts of Interest

K.B. and S.C.D.S. declare financial interest in the company Trince.

## Supporting information




**Supporting File 1**: advs77816‐sup‐0001‐SuppMat.docx.

**Supporting File 2**: advs77816‐sup‐0002‐VideoS1.mp4.

## Data Availability

The data that supports the findings of this study are available in the supplementary material of this article.
