# Supplementary material for: Detaching Photosensitive Nanoparticles from Cells Prevents Them from Remaining in Cells after Photoporation
Source: Adv Sci (Weinh). 2026 Sep 25:e77816. Online ahead of print. doi: 10.1002/advs.77816 (PMC13616249; doi:10.1002/advs.77816)
Supplement: Supplementary file 1 [file ADVS-9999-e77816-s001.docx]

**Supporting Information**

**Detaching Photosensitive Nanoparticles from Cells Prevents Them from Remaining in Cells after Photoporation**

*Tao Lu^1,2^, Mina Nikolić^3^, Baihao Huang^1^, Deep Punj^2^, Herlinde De Keersmaecker^2,4^, Thibaut Van Acker^3^, Wenjia Xie^2^, Florian Vanlauwe^5^, Phillip Blondeel^5^, Liesl De Graeve^6^, Félix Sauvage^2^, Ranhua Xiong^1^, Chaobo Huang^1^, Frank Vanhaecke^3^, Kevin Braeckmans ^1,2,4^, Stefaan C. De Smedt^1,2^**

^1^ State Key Laboratory for Development and Utilization of Forest Food Resources, Joint Laboratory of Advanced Biomedical Materials (NFU-UGent), Jiangsu Co-Innovation Center of Efficient Processing and Utilization of Forest Resources, Nanjing Forestry University, Nanjing 210037, China

^2^ Laboratory of General Biochemistry and Physical Pharmacy, Faculty of Pharmaceutical Sciences, Ghent University, Ghent 9000, Belgium

^3^ Atomic and Mass Spectrometry – A&MS research group, Department of Chemistry, Faculty of Sciences, Ghent University, Ghent 9000, Belgium

^4^ Ghent Light Microscopy Core, Ghent University, Ghent 9000, Belgium.

^5^ Tissue Regeneration and Organ Printing (TROP) Research Center, Department of Human Structure and Repair, Ghent University, Ghent, Belgium

^6^ Department of Plastic and Reconstructive Surgery, Ghent University Hospital, Ghent, Belgium

*Corresponding author. E-mail: [stefaan.desmedt@ugent.be](mailto:stefaan.desmedt@ugent.be)

# Supporting information

# 1. Supporting notes

## 1.1 Determination of the vapor nanobubble generation threshold for AuNPs

To precisely determine this threshold and characterize the VNB generation dynamics, the experimental data were fitted using a Boltzmann sigmoidal model:

$y=A_{2}+\frac{A_{1}-A_{2}}{1+exp((x-x_{0})/dx)}$	(1)

In this equation, $A_{0}$and $A_{1}$ represent the lower and upper plateaus of the VNB count, respectively, while $x_{0}$ denotes the inflection point (midpoint) of the transition and $d_{x}$ signifies the width of the response curve. The VNB generation threshold $x_{90}$ was then calculated as $x_{90}=x_{0}+d_{x}\cdot\ln(9)$, representing the laser fluence required to reach 90% of the saturation plateau.

**1.2 Delivery yield**

To evaluate the overall performance of the photoporation process, the delivery yield was determined by integrating the delivery (or transfection) efficiency with cell viability data obtained from flow cytometry. This parameter represents the fraction of the initial cell population that was both successfully transfected and remained viable. The delivery yield was calculated according to the following equation:

$Delivery yield [\%]=\frac{Delivery efficiency [\%]-Cell viability [\%]}{100}$	(2)

**1.3 Quantitative Analysis of Intracellular Delivery and Expression (rMFI)**

To quantify the amount of internalized cargo (e.g., FD500) or the level of protein expression (e.g., eGFP) per cell, the relative mean fluorescence intensity (rMFI) was calculated from flow cytometry data. Three distinct populations were analyzed: untreated control cells ($I_{U}$), uptake control cells (cells incubated with cargo but not laser-treated, $I_{C}$), and the photoporated sample ($I_{P}$). All measured intensities were normalized against the mean autofluorescence of the untreated cells ($I_{U}$) to ensure consistent comparisons across different experiments and instrument settings.

The rMFI of the entire photoporated population (rMFI_pos_) was first determined as the difference between the sample and uptake control intensities relative to the untreated control:

${rMFI}_{pop}=\frac{I_{P}-I_{C}}{I_{U}}$	(3)

To specifically evaluate the payload concentration within successfully treated cells, the rMFI of the positive cell population (rMFI_pos_) was calculated using the following formula:

${rMFI}_{pos}=\frac{\frac{I_{P}-I_{C}}{I_{U}}-f_{P-}\cdot\frac{I_{P-}-I_{C-}}{I_{U}}}{f_{P+}}$	(4)

Where $f_{P+}$ represents the transfection efficiency (percentage of positive cells) and $f_{P-}$ is the fraction of negative cells, both determined by applying a 95% threshold based on the uptake control distribution. $I_{P-}$ and $I_{C-}$ denote the mean intensities of cells falling below the selected threshold in the photoporated and uptake control samples, respectively. This calculation ensures that rMFI_pos_ accurately reflects the relative fluorescence contribution solely from the cells that successfully underwent photoporation.

## 1.4 Isolation and culture of AD-MSCs

Human adipose-derived mesenchymal stromal cells (AD-MSCs) were mechanically isolated from abdominal lipoaspirates using a previously reported inter-syringe processing method with minor modifications.^[1-2]^ Human adipose tissue was obtained from donors undergoing liposuction at Ghent University Hospital. The use of lipoaspirate for in vitro tissue culture and characterization received positive ethical advice from the Medical Ethics Committee of Ghent University and Ghent University Hospital (reference no. ONZ-2025-0106; 12 May 2025). Written informed consent was obtained from all donors using the committee-approved informed consent form. Briefly, freshly collected lipoaspirate was transferred into 10 mL Luer-Lok™ syringes and centrifuged at 3000 rpm for 1 min. Following centrifugation, the upper oily phase and lower aqueous phase were discarded, while the intermediate adipose tissue fraction was retained. A 10 mL syringe containing 5 mL of the centrifuged adipose tissue was connected through a Luer-to-Luer connector to another 10 mL syringe containing 5 mL of sterile phosphate-buffered saline (PBS). The contents were forcefully passed between the two syringes 30 times to mechanically emulsify the adipose tissue. The resulting PBS-diluted emulsified tissue was pooled and centrifuged at 3000 rpm for 10 min. The cell-containing pellet was collected, resuspended in complete culture medium (CM), and seeded into tissue-culture flasks. After 48 h, the medium was replaced to remove non-adherent cells.

Complete CM consisted of MesenCult™ MSC Basal Medium (Human) supplemented with 10% (v/v) MesenCult™-hPL 10X Supplement (STEMCELL Technologies, Vancouver, Canada), 1% (v/v) penicillin–streptomycin, and 0.5% (v/v) amphotericin B (both from Thermo Fisher Scientific, Waltham, MA, USA). Cells were maintained at 37 °C in a humidified atmosphere containing 5% CO₂, and the culture medium was replaced every 3 days. Upon reaching approximately 90% confluence, the cells were detached using TrypLE™ Express (Thermo Fisher Scientific) for 5 min and passaged at a 1:2 split ratio. AD-MSCs at passages P1–P5 were used for the experiments.

# 2. Supporting video

## 2.1 Supporting Video 1: Time-Resolved Visualization of VNB Dynamics

Under dark-field microscopy, VNB formation was visualized as transient, bright scattering signals (Figure 2f), originating from the rapid localized heating of the AuNPs, which triggers the thermal expansion and subsequent implosion of vapor cavities.

To resolve these rapid kinetics, high-speed imaging was captured using a sCMOS camera at a temporal resolution of 1,000 frames per second (FPS), allowing for a frame-by-frame analysis of the transient VNB life cycle **(Figure S2a,b)**. As demonstrated in **Supporting Video 1**, the recorded sequence is presented at a 1000-fold slower speed to facilitate the observation of these microsecond-scale dynamics. The results confirm that VNBs are triggered immediately upon laser irradiation; while the actual physical event occurs on a sub-millisecond scale, the scattering signal typically emerges and dissipates within a single 1 ms frame window at this sampling rate.

# 3. Supporting figures

**Figure S1.** Chemical structures of the reagents used in this study. Polyethylenimine (PEI), phenylboronic acid (PBA), 4-mercaptophenylboronic acid (MPBA) and adenosine triphosphate (ATP).

**Figure S2.** **Time-resolved dark-field microscopy images capturing the formation and collapse of VNBs following a single nanosecond laser pulse.** **(a)** A representative time-lapse dark-field image sequence illustrating the transient formation of vapor nanobubbles (VNBs). The sequence captures the complete dynamic cycle of the VNB (1–5 ms post-irradiation), spanning from initial nucleation and rapid expansion to the final collapse. Scale bar is 10 μm. **(b)** Temporal profile of the relative scattering intensity (represented by mean gray value (MGV)) during VNB formation and collapse. The MGV provides a real-time trace of the bubble kinetics; the peak intensity within 1 ms denotes the maximum scattering cross-section, while the subsequent abrupt decline signifies the rapid bubble collapse and dissipation.

**Figure S3.** **FD500 delivery performance of citrate-Au NPs as a function of laser fluence. (a)** Delivery performance at a citrate-AuNPs concentration of 50 × 10^7^ NPs/mL. **(b)** Delivery performance at a citrate-AuNPs concentration of 100 × 10^7^ NPs/mL. From left to right: (i) FD500 delivery efficiency (percentage of FITC-positive cells) and corresponding cell viability; (ii) the relative mean fluorescence intensity of positive cells (rMFI_pos_); and (iii) the delivery yield. Data are presented as mean ± SD (N=3, n=3). **(c)** 3D response surface plots illustrating the optimization of delivery yield as a function of AuNP concentration (X-axis, NPs mL^−1^) and laser fluence (Y-axis, J cm^−2^). The Z-axis represents the delivery yield (%).

**Figure S4.** **FD500 delivery performance of PBA-PEI/AuNPs as a function of laser fluence. (a)** Delivery performance at a PBA-PEI/AuNPs concentration of 50 × 10^7^ NPs/mL. **(b)** Delivery performance at a PBA-PEI/AuNPs concentration of 100 × 10^7^ NPs/mL. From left to right: (i) FD500 delivery efficiency (percentage of FITC-positive cells) and corresponding cell viability; (ii) the relative mean fluorescence intensity of positive cells (rMFI_pos_); and (iii) the delivery yield. Data are presented as mean ± SD (N=3, n=3).

**Figure S5.** **Optimization of photoporation mediated FD500 delivery into Jurkat cells using MPBA-functionalized AuNPs**. **(a)** Delivery efficiency (left Y-axis) and cell viability (right Y-axis) of Jurkat cells treated with MPBA-AuNPs modified at different MPBA concentrations (as used in the coating of the AuNPs). For photoporation 100 × 10^7^ NPs mL^−1^ and a laser fluence of 1.2 J cm^−2^ were used. **(c)** Effect of laser fluence at a fixed concentration of 50 × 10^7^ NPs/mL. **(d)** Effect of laser fluence at a fixed concentration of 100 × 10^7^ NPs/mL. From left to right: (i) FD500 delivery efficiency (percentage of FITC-positive cells) and corresponding cell viability; (ii) the relative mean fluorescence intensity of positive cells (rMFI_pos_); and (iii) the delivery yield. Data are presented as mean ± SD (N=3, n=3).

**Figure S6. Optimization of photoporation mediated eGFP-mRNA delivery into Jurkat cells using MPBA-functionalized AuNPs.** (a) Corresponding relative mean fluorescence intensity (rMFI) of eGFP-positive cells. (b) Transfection yield (%) calculated from rMFI and viability data. Data are presented as mean ± SD (N=3, n≥3).

**Figure S7. Detachment of AuNPs from the cell surface.** Citrate-Au NPs (control) and PBA-PEI/AuNPs on HeLa cells before are after extra washing with cell culture medium (CCM) or 4 mM ATP. Scale bar is 10 μm.

## Confirming attachment/detachment of AuNPs by spatially selective delivery of AF10 dextran

To evaluate the detachment efficiency of MPBA-AuNPs from the cell surface, spatially selective intracellular delivery of Alexa Fluor™ 488-labeled dextran (10,000 MW, AF10) was performed. HeLa cells were seeded at a density of 1 × 10^4^ cells/well in 96-well plates and allowed to adhere overnight until reaching approximately 90% confluency. The following day, the cells were incubated with MPBA-functionalized AuNPs for 10 min to allow for attachment. To investigate the impact of different washing conditions on AuNPs retention and subsequent delivery, the cells were divided into four experimental groups: (1) The no-treatment control group (NTC), consisting of cells without nanoparticles and laser treatment; (2) MPBA-AuNPs (No wash): No additional washing steps were performed after NP incubation; (3) MPBA-AuNPs (CCM wash): After removing unbound NPs, the cells were rinsed with standard cell culture medium (CCM); (4): MPBA-AuNPs (4 mM ATP wash): After removing unbound NPs, the cells were washed three times with CCM supplemented with 4 mM ATP.

Following the respective washing procedures, an AF10 solution (0.1 mg mL^-1^) was added to each well. For laser treatment, the plates were placed on an electronic motorized translation stage (H117, Prior, UK), which facilitated automated laser scanning across each well **(Figure S8)**. The laser beam followed a serpentine (snake-like) pattern at a constant scanning speed of 7 × 10^3^ µm s^−1^. To ensure continuous and comprehensive coverage of the entire well surface without overlap or omission, the beam moved line-by-line with a fixed step width of 0.3 mm in the Y-direction, precisely matching the laser spot diameter. The scanning dimensions (X- and Y-fields) were pre-defined to control the number of scan lines, while the edge of the 96-well plate served as a spatial reference point to initialize each sequence. This automated setup guaranteed uniform laser exposure across the entire well area.

After irradiation, the cells were stained with Calcein-AM for live-cell visualization and observed via confocal microscopy. In the resulting images, green fluorescence indicated viable cells, while red fluorescence was used to represent successfully delivered AF10 molecules.

**Figure S8.** **Confirming attachment/detachment of AuNPs by spatially selective delivery of AF10 dextran.** **(a)** Schematic illustration of pulsed laser scanning strategy used for patterned photoporation. **(b)** Example of a “snake-like” laser scanning pattern applied across the cell monolayer to generate a predefined pattern.

**Figure S9.** **Workflow of reversible PBA-AuNP-mediated photoporation for intracellular delivery and subsequent analysis.**

**Figure S10.** **Effect of short-term ATP exposure on Jurkat cell recovery and Caspase-3/7 activity.** Jurkat cells were exposed to the indicated ATP concentrations (0-16 mM) for 5 min. The ATP-containing medium was subsequently removed, and the cells were resuspended in fresh RPMI 1640 medium and cultured for 24 h. **(a)** Cell recovery after 24 h, expressed relative to the mean value of the untreated control group (0 mM ATP). **(b)** Total Caspase-3/7 luminescence per sample, expressed relative to the untreated control. **(c)** Caspase-3/7 luminescence normalized to the corresponding number of recovered cells and subsequently expressed relative to the untreated control. Data represent the mean ± SD of three independent experiments (N = 3).

**Figure S11. 7-day proliferative capacity of Jurkat cells following ATP treatment, AuNP incubation, laser irradiation, and photoporation.** **(a)** Jurkat cells were subjected to the indicated treatments and subsequently seeded at an equal density of 4,000 cells per well. The experimental conditions included untreated cells (NTC), ATP treatment alone, laser irradiation alone, incubation with 1 × 10^9^ particles mL^-1^ AuNPs without laser irradiation, and photoporation with either 5 × 10^8^ or 1 × 10^9^ particles mL^-1^ AuNPs. Following laser irradiation, both photoporation groups were subjected to ATP-mediated AuNP detachment using 4 mM ATP for 5 min. Cell densities were determined on days 1, 3, 5, and 7 after treatment. Each sample had a total culture volume of 1.2 mL. **(b)** Cell density during the 7-day follow-up period. The cell-density axis is presented on a base-2 logarithmic scale to visualize successive increases across the complete measurement range. Lines represent group means, error bars indicate SD, and individual points represent independent experiments. **(c)** Cell density on day 7. Data represent the mean ± SD of three independent experiments (N = 3).

**Figure S12. Viability and Caspase-3/7 activity of adipose-derived mesenchymal stromal cells (AD-MSCs) following photoporation and ATP-mediated AuNP detachment. (a)** Schematic illustration of cell-surface attachment of PBA-functionalized AuNPs, laser irradiation, and subsequent ATP-mediated AuNP detachment. **(b–d)** Short-term responses of adipose-derived mesenchymal stromal cells (AD-MSCs) measured 24 h after the indicated treatments. The experimental groups included non-treated cells (NTC), ATP treatment alone, laser irradiation alone, incubation with 1 × 10^9^ particles mL^-1^ AuNPs without laser irradiation, and photoporation using either 5 × 10^8^ or 1 × 10^9^ particles mL^-1^ AuNPs. Following laser irradiation, both photoporation groups were washed with 4 mM ATP for 5 min to induce AuNP detachment. **(b)** CTG-derived metabolic viability expressed relative to the mean luminescence of the NTC group. **(c)** Total Caspase-3/7 luminescence per sample expressed relative to the NTC group. **(d)** Caspase-3/7 luminescence normalized to the corresponding CTG signal and subsequently expressed relative to the NTC group. **(e)** Experimental workflow for assessing AD-MSC proliferation over 7 days following treatment. After treatment, the cell density was adjusted to 2 × 10^4^ cells mL⁻¹, and 4,000 cells were seeded per well. **(f)** Proliferation profiles determined by cell counting on days 1, 3, 5, and 7. The cell-density axis is presented on a logarithmic scale. **(g)** Cell density of the treatment groups on day 7. **(h)** CTG-evaluated metabolic viability on day 7, normalized to the mean signal of the NTC group. Data represent the mean ± SD of three independent experiments (N = 3).

# References

1.	L. Benmeridja, L. De Moor, E. De Maere, et al.,“High-throughput fabrication of vascularized adipose microtissues for 3D bioprinting,” *Journal of Tissue Engineering and Regenerative Medicine*, 14 (2020): 840.

<https://doi.org/https://doi.org/10.1002/term.3051>

2.	M. Doornaert, E. De Maere, J. Colle, et al.,“Xenogen-free isolation and culture of human adipose mesenchymal stem cells,” *Stem Cell Research*, 40 (2019): 101532.

<https://doi.org/https://doi.org/10.1016/j.scr.2019.101532>
